# *PIK3CA* mutational status in tissue and plasma as a prognostic biomarker in HR+/HER2− breast cancer

**DOI:** 10.1002/cam4.70101

**Published:** 2024-09-05

**Authors:** Eduardo Terán, Rebeca Lozano, César A. Rodríguez, Mar Abad, Luis Figuero, José Antonio Muñoz, Belén Cigarral, Aline Rodrígues, Magdalena Sancho, M. Asunción Gómez, Daniel Morchón, Juan Carlos Montero, José María Sayagués, M. Dolores Ludeña, Emilio Fonseca

**Affiliations:** ^1^ Medical Oncology Department University Hospital of Salamanca Salamanca Spain; ^2^ Institute of Biomedical Research of Salamanca (IBSAL) Salamanca Spain; ^3^ Pathology Department University Hospital of Salamanca Salamanca Spain; ^4^ Biomedical Research Networking Centers‐Oncology (CIBERONC) Madrid Spain

**Keywords:** ctDNA, cyclin inhibitors, luminal breast cancer, *PIK3CA*, tissue

## Abstract

**Introduction:**

Hotspots (HS) mutations in the *PIK3CA* gene may lead to poorer oncological outcomes and endocrine resistance in advanced breast cancer (BC), but their prognostic role in early‐stage disease remains controversial. The overall agreement within plasma and tissue methods has not been well explored. Our aim was to correlate tissue and plasma approaches and to analyze the prognostic impact of *PIK3CA* mutations (*PIK3CAm*) in HR+/HER2− BC.

**Methods:**

A retrospective and unicentric analysis of *PIK3CA* mutational status in tissue and plasma samples by Cobas®PIK3CA Mutation Kit in patients with HR+/HER2− BC.

**Results:**

We analyzed 225 samples from 161 patients with luminal BC. *PIK3CA* mutations were identified in 62 patients (38.5%), of which 39.6% were found in tissue and 11.8% in plasma. In advanced disease, plasma and tissue correlation rate was performed in 64 cases, with an overall agreement of 70.3%. Eighty patients were treated with CDK4/6 inhibitors + endocrine therapy. We observed a moderately worse progression‐free survival (PFS) in *PIK3CAm* versus wild‐type (WT) (24 m vs. 30 m; HR = 1.39, *p* = 0.26). A subanalysis was carried out based on exons 9 and 20, which showed a statistically poorer PFS in *PIK3CAm* exon 9 versus 20 population (9.7 m vs. 30.3 m; HR = 2.84; *p* = 0.024). Furthermore, detection of *PIK3CAm* in plasma was linked to a worse PFS vs *PIK3CAm* detection just in tissue (12.4 vs. 29.3; HR = 2.4; *p* = 0.08).

**Conclusions:**

Our findings suggest the *PIK3CA* evaluation in tissue as the diagnostic method of choice, however, additional investigations are required to improve the role of liquid biopsy in the *PIK3CA* assessment. *PIK3CAm* show worse outcomes in advanced luminal BC, especially in exon 9 mutation carriers, despite visceral involvement, prior exposure to endocrine therapy or detection of *PIK3CAm* in plasma, with an unclear prognosis in early‐stage disease. Nonetheless, this should be validated in a prospective cohort study.

## Graphical abstract

*PIK3CA* gene assessment in tissue remains the main diagnostic approach. The use of highly sensitive techniques or the timing of peripheral blood collection could better characterize the contribution of liquid biopsy in the identification of *PIK3CA* mutations. Despite the limited sample size, *PIK3CAm* may lead to worse outcomes in advanced luminal breast cancer (BC), particularly showing a significantly poorer prognosis in exon 9 mutation carriers, independent of visceral involvement, and plasma detection. However, their impact remains unclear in early‐stage BC, and these findings need confirmation in a larger prospective and separate cohort.

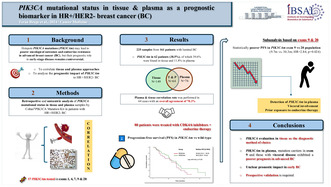

## INTRODUCTION

Phosphatidylinositol 3‐kinases (PI3Ks) play a critical role in several processes involved in tumor progression, such as growth, cell metabolism, and survival through the PI3k/AKT/mTOR pathway.^1^, ^2^, ^3^ Multiple investigations have revealed that this route is upregulated in approximately 60%–75% of tumors, including breast cancer (BC).^4^, ^5^

Activating mutations in the *PIK3CA* (*PIK3CAm*) gene directly alter the function of the PI3 kinase protein, causing abnormal activation of the PI3 kinase signaling pathway and promoting cancer progression. In contrast, neutral somatic mutations are common variants found in the general population that have minimal impact on protein function.^6^

Hotspot (HS) mutations in *PIK3CA* are detected in approximately 30%–40% of luminal BCs,^7^ resulting in hyperactivation of the catalytic subunit (p110α) of phosphatidylinositol‐3‐kinase (PI3K). *PIK3CAm* most commonly occur at three HS sites: E542K and E545K in exon 9 (helical domain), and H1047R in exon 20 (kinase domain).^8^

The implications of *PIK3CAm* and patient outcomes remain controversial. Some studies have suggested poorer clinical outcomes in advanced disease, resulting in lower overall survival (OS) due to the constitutive activation of the PI3K/AKT/mTOR pathway, which promotes cell growth and invasion, and increased resistance to endocrine therapy, chemotherapy, and some targeted therapies in *PIK3CAm* carriers.^9^, ^10^

On the other hand, *PIK3CAm* in early BC (eBC) has also been reported by some authors to be associated with increased recurrence‐free survival^11^ and responsiveness to hormonal therapy. Although its prognostic role in early stage is more debated^12^, ^13^ and may vary based on clinical circumstances and the specific treatment regimen employed.

Recently, the prognostic impact of *PIK3CAm* in patients treated with cyclin inhibitors has been reported,^14^ identifying the presence of *PIK3CAm* as a promising predictive biomarker of resistance to CDK4/6 inhibitors.

Based on the phase III trial, SOLAR‐1,^15^ *PIK3CAm* have reached level 2 evidence for predicting benefit, according to the Magnitude of Clinical Benefit Scale by ESMO, from fulvestrant combined with alpelisib, an alpha‐specific PI3K inhibitor in patients with advanced HR+/HER2− BC, who had previously progressed to a previous line of endocrine therapy, showing a significantly higher median PFS compared to placebo plus fulvestrant (HR = 0.65 [95% CI, 0.50–0.85], *p* < 0.001). Hence, with the recent approval of alpelisib for BC patients whose tumors harbor *PIK3CAm*, the proficiency testing for *PIK3CAm* analysis is essential.

In addition, plasma‐derived circulating tumor DNA (ctDNA) offers real‐time insight into genetic alterations and a non‐invasive view of tumor heterogeneity. The determination of *PIK3CAm* in plasma could provide critical information and discriminate patients who would benefit from treatment with *PIK3CA* inhibitors. Also, the plasma detection rate of *PIK3CAm* in BC is highly variable, ranging from 26% to 93%,^16^ and correlation rate among testing methods (tissue and plasma) has not been well explored. Our aim was to correlate tissue and plasma approaches and to analyze the prognostic impact of *PIK3CA* mutations (*PIK3CAm*) in HR+/HER2− BC.

## 1 METHODS

### 1.1 Study design and participants

This is a retrospective, unicentric, and observational analysis of *PIK3CA* mutational status in tissue and plasma in patients with HR+/HER2− BC from February 2021 to April 2023 in the University Hospital of Salamanca (HUSA).

Samples obtained from patients with HR+/HER2− BC were collected from tissue (primary tumor or metastases) and plasma. Liquid biopsy was obtained exclusively from patients with metastatic disease. The patients met the following criteria: histopathological confirmed diagnosis of BC, with complete clinical and histological data.

### 1.2 Technical analysis of *PIK3CA* in plasma and tissue

DNA extraction from formalin‐fixed paraffin‐embedded (FFPE) tumor tissue and liquid biopsy was conducted using Cobas® DNA/cfDNA sample preparation kit (Roche Diagnostics), according to the manufacturer's guidelines. A comprehensive analysis of the technical process of tissue and plasma sample extraction is included in the Data S1.

The DNA obtained from FFPE and liquid biopsy samples were used to analyze 17 mutations across exons 1, 4, 7, 9, and 20 of the *PIK3CA* gene (Roche Diagnostics) using the Cobas® PIK3CA Kit (Cobas® platform, Roche Diagnostics).^17^

The Cobas® PIK3CA Mutation Test platform is specifically designed to differentiate activating mutations in the *PIK3CA* gene from neutral somatic mutations and germline polymorphisms. It focuses on recognized mutation HS within *PIK3CA* (associated with functional changes in the PI3 kinase protein, which can lead to abnormal activation of the PI3 kinase signaling pathway), utilizing an extensive database of validated mutations, rigorous quality control measures, and real‐time PCR technology for targeted amplification and sequencing of pertinent gene regions. Detailed clinical reports are generated to provide precise information for making informed therapeutic decisions based on the mutation profile identified in the patient's *PIK3CA* gene.^17^

### 1.3 Study outcomes

Clinical assessment criteria, such as response rate, disease‐free survival (DFS), or metastasis‐free interval and progression‐free survival (PFS) were analyzed.

We correlated both diagnostic methods (tissue and plasma) and examined the discordant cases and patient characteristics. We analyzed clinical outcomes in *PIK3CAm* and wild‐type (WT) populations in terms of metastasis‐free interval and PFS during treatment with CDK4/6 inhibitors, according to HS mutations (exon 9 vs. 20), visceral involvement, metastatic sites, and detection in plasma.

### 1.4 Statistical analysis

A descriptive analysis of the clinicopathological features of the population was performed according to *PIK3CA* status (*PIK3CAm* vs. WT). To assess the quality of the statistical data for group differentiation, we used Student's *t*‐test and Mann–Whitney *U*‐test for normally and abnormally distributed continuous variables, respectively. SPSS v.22 (IBM Corp., Armonk, NY, USA) was applied to calculate continuous variables and measures of central tendency, such as mean and standard deviation.

Frequencies and percentages were indicated as dichotomous variables using Pearson's *X* ^2^ test. The statistical significance of the differences in survival was determined by the Mantel–Cox test (log‐rank test) and the Kaplan–Meier method. Cox regression models were used to calculate the hazard ratios (HRs) and 95% confidence intervals (CIs) for PFS. Multivariate analysis of prognostic factors for PFS was carried out using multivariate Cox regression models. Results were defined as significant when two‐sided *p*‐values were <0.05.

## 2 RESULTS

### 2.1 Mutational spectrum

We included 161 patients, 71 with eBC and 90 patients with advanced disease. A total of 225 samples from 161 patients were analyzed: 149 tissue samples and 76 plasma samples. Mutations in the *PIK3CA* gene (*n* = 69) were identified in 62 patients (38.5%). Of these, 39.6% were in tissue (59 patients) and 11.8% were in plasma (9 patients). Seven patients (11.3%) had >1 activating mutation in the *PIK3CA* gene. Plasma was obtained exclusively from patients with advanced disease.

The most frequent *PIK3CAm* were those located in the three HS: the tyrosine kinase domain of exon 20; H1047X (44.9%), followed by mutations in the helical domain of exon 9 (37.7%), E545X (20%), E542K (13%), and Q546X (4.7%). Rare mutations in exon 7 were described in 10.1% (C420R) and in exon 4 in 7.2% (N345K).

We reported a similar *PIK3CA* mutational rate in eBC; 38% (27 out of 71 patients) and in advanced disease; 38.8% (35 out of 90 patients), OR = 0.96 (95% CI, 0.51–1.83); *p* = 0.91. Furthermore, according to the site of *PIK3CAm* determination, analysis was performed on primary tumor tissue in 121 subjects (81.2%) and on metastatic tissue in 28 patients (18.8%), including hepatic (*n* = 12), pulmonary (*n* = 4), soft tissue (*n* = 6), lymph node (*n* = 4), ovarian (*n* = 1), and adrenal (*n* = 1). We did not observe a significantly higher proportion of *PIK3CAm* in primary tumor tissue versus metastases [40.5% vs. 35.7%, OR = 1.23 (95% CI, 0.52–2.88); *p* = 0.64].

### 2.2 Plasma–tissue correlation analysis

We conducted a plasma–tissue correlation study in 64 metastatic patients for whom paired samples were available. We found *PIK3CAm* in 28 (43.8%). The overall tissue–plasma correlation rate was 70.3%, with a positive agreement in 9 of 28 patients (32.1%). Interestingly, a discordant result was observed in 19 cases (29.7%) with the detection of *PIK3CAm* in tissue but not in plasma, as shown in Table 1.

**TABLE 1:** Tissue and plasma correlation analysis of *PIK3CAm* carriers.

	Tissue		
	Mutated	WT	Total
Plasma			
Mutated	**9 (32.1%)**	0 (0%)	9 (14.1%)
WT	19 (67.8%)	**36 (100%)**	55 (85.9%)
Total	28 (100%)	36 (100%)	64 (100%)
	**Positive agreement: 9/28 (32.1%)**		
	**Negative agreement: 36/36 (100%)**		
	**Overall agreement: 45/64 (70.3%)**		

Bold values indicate that 9 (32.1%) of cases have mutations detected in both tissue and plasma, demonstrating a positive correlation. Meanwhile, 36 cases (100%) showed no mutations in either tissue or plasma, indicating complete negative concordance.

For patients with paired tissue‐plasma studies (*n* = 64), *PIK3CA* determination was performed in primary tumor tissue for 60.9% and in metastases for 39.1%. We observed a similar proportion of *PIK3CAm* in primary tumor tissue or metastases [41% vs. 36%, OR = 1.23 (95% CI, 0.44–3.49); *p* = 0.69].

The timing of the samples was characterized by the median time (in days) between tissue biopsy and plasma collection. For the overall population, we observed a median interval of 779.5 days with an interquartile range (IQR) of 1150. In patients with *PIK3CAm*, this interval extended to 1256 days with an IQR of 2309, while for those with *PIK3CA* WT, it was 721 days with an IQR of 954. We found no significant association between the timing of sample collection and the presence of *PIK3CAm*; *p* = 0.54.

We conducted an analysis to identify the characteristics associated with the *PIK3CAm* detection in plasma. *PIK3CAm* detection in plasma was more frequent in population with ≥3 metastatic sites [66.7% vs. 31.6%, OR = 4.33 (95% CI, 0.79–23.5); *p* = 0.08], visceral involvement [66.7% vs. 47.4%, OR = 0.53 (95% CI, 0.91–3.14); *p* = 0.43] and sample collection during disease progression [66.7% vs. 47.4%, OR = 2.22 (95% CI, 0.43–11.60); *p* = 0.44]. Specifically, *PIK3CAm* was detected in plasma in 50% of patients with ≥3 metastatic sites and in 40% of patients with visceral involvement. Also, it should be noted that plasma collection during disease progression was performed in 54.7% of patients (35 out of 64 patients).

### 2.3 Patient characteristics

Clinicopathological characteristics are listed in Table 2. One hundred sixty‐one patients were included, with a median age of 54 years [21–91]. Seventy‐one patients with eBC and 90 patients with advanced disease were analyzed. According to histological subtype, we observed a significantly higher proportion of BC with lobular histology in *PIK3CAm* carriers versus WT [18.1% vs. 4.1%, OR = 5.12 (95% CI, 1.55–16.89); *p* = 0.007]. Other clinicopathological features reflected in Table 2 showed a similar proportion between both groups (*PIK3CAm* vs. WT). Smaller tumors (<50 mm), less nodal involvement, lower Ki‐67 index, and histological grade were observed in *PIK3CAm* versus WT population, although not statistically significant.

**TABLE 2:** Clinical and pathological features of carriers and non‐carriers of *PIK3CAm* in our sample.

		*PIK3CAm*	*PIK3CA* WT	*p*‐value
		(*n* = 62)%	(*n* = 99)%	
Age; median [min‐max]		55 [24–90]	56 [33–91]	
Histological type	Ductal	47 (77%)	81 (83.5%)	***p* = 0.007**
	Lobular	11 (18.1%)	4 (4.1%)	
	Others	3 (4.9%)	12 (12,4%)	
Histological grade	1	18 (31.6%)	15 (16.1%)	*p* = 0.06
	2	33 (57.9%)	62 (66.7%)	
	3	6 (10.5%)	16 (17.2%)	
Ki67 (%)	<14%	31 (53.4%)	42 (44.2%)	*p* = 0.26
	≥14%	27 (46.6%)	53 (55.8%)	
Her2	Negative/0	42 (72.4%)	57 (60.6%)	*p* = 0.13
	1+/2+	16 (27.6%)	37 (39.4%)	
Tumor size	<50 mm	42 (73.7%)	79 (82,3%)	*p* = 0.21
	≥50 mm	15 (26.3%)	17 (17.7%)	
Nodes (*N*)	N0	19 (37.3%)	30 (33.3%)	*p* = 0.63
	N+	32 (62.7%)	60 (66.7%)	
Debut M1	Yes	13 (37.1%)	21 (38.2%)	*p* = 0.92
	No	22 (62.9%)	34 (61.8%)	
Visceral involvement	Yes	26 (74.3%)	40 (72.7%)	*p* = 0.87
	No	9 (25.7%)	15 (27.3%)	

*Note*: M1: advanced disease.

Abbreviation: WT, wild‐type.

Bold values indicate a significant association between PIK3CAm and the histological type of breast cancer. Notably, patients with PIK3CAm exhibit a higher prevalence of lobular histology (18.1%) compared to those without the mutation (4.1%). This difference is statistically significant, as indicated by a *p*‐value of 0.007.

### 2.4 Prognostic value of *PIK3CAm*

In the overall population assessed for DFS (*n* = 127); 78 patients were *PIK3CAm* (61.4%) and 49 were *PIK3CA* WT (38.6%), excluding those with advanced stage at onset. During follow‐up, 56 patients developed metastatic disease, while 71 did not experience recurrences.

**FIGURE 1:**
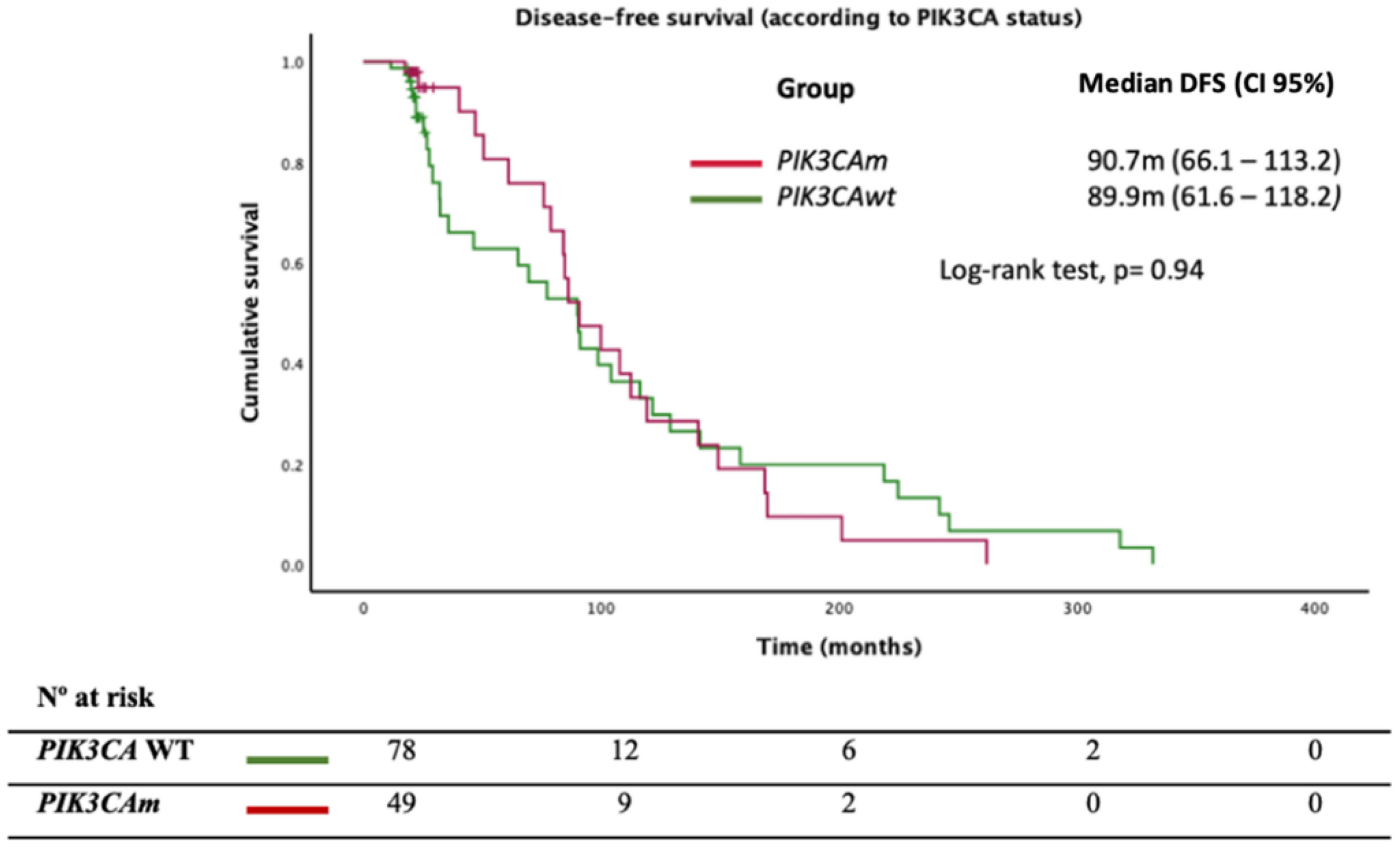
Disease‐free survival (DFS) according to the molecular status of *PIK3CA* gene.

Among the 78 *PIK3CA* WT patients, 44 (56.4%) did not experience recurrence, compared to 27 out of 49 *PIK3CAm* patients (55.1%). We did not observe a higher proportion of recurrences in the *PIK3CAm* population compared to WT [44.9% vs. 43.6%; *p* = 0.89, OR = 1.05 (95% CI, 0.51–2.17)]. Excluding patients who did not experience recurrences (censored cases), we observed similar DFS in the *PIK3CAm* vs WT population [90.7 m vs. 89.9 m, HR = 0.98 (95% CI, 0.56–1.69); *p* = 0.94], as illustrated in Figure 1.

Regarding HS mutations, although not statistically significant, we observed longer DFS in the *PIK3CAm* exon 20 compared to exon 9 population [112.4 m vs. 107.7 m, HR = 1.67 (95% CI, 0.57–4.91); *p* = 0.35].

**FIGURE 2:**
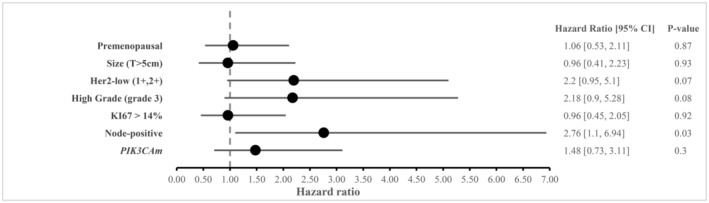
Multivariate analysis evaluating the prognostic impact in terms of DFS of clinical and molecular variables in eBC patients.

Univariate and multivariate analysis were conducted to evaluate the impact of *PIK3CAm* mutational status on DFS in eBC patients. Multivariate analysis in Figure 2, identified nodal involvement as an independent prognostic factor for DFS in eBC [HR = 2.76 (95% CI, 1.1–6.94); *p* = 0.03].

Additionally, we reported shorter DFS in patients with HER2− low [HR = 2.2 (95% CI, 0.95–5.1); *p* = 0.07] and grade 3 tumors [HR = 2.18 (95% CI, 0.9–5.28); *p* = 0.08].

#### 2.4.1 *PIK3CA* evaluation in population treated with cyclin inhibitors

**FIGURE 3:**
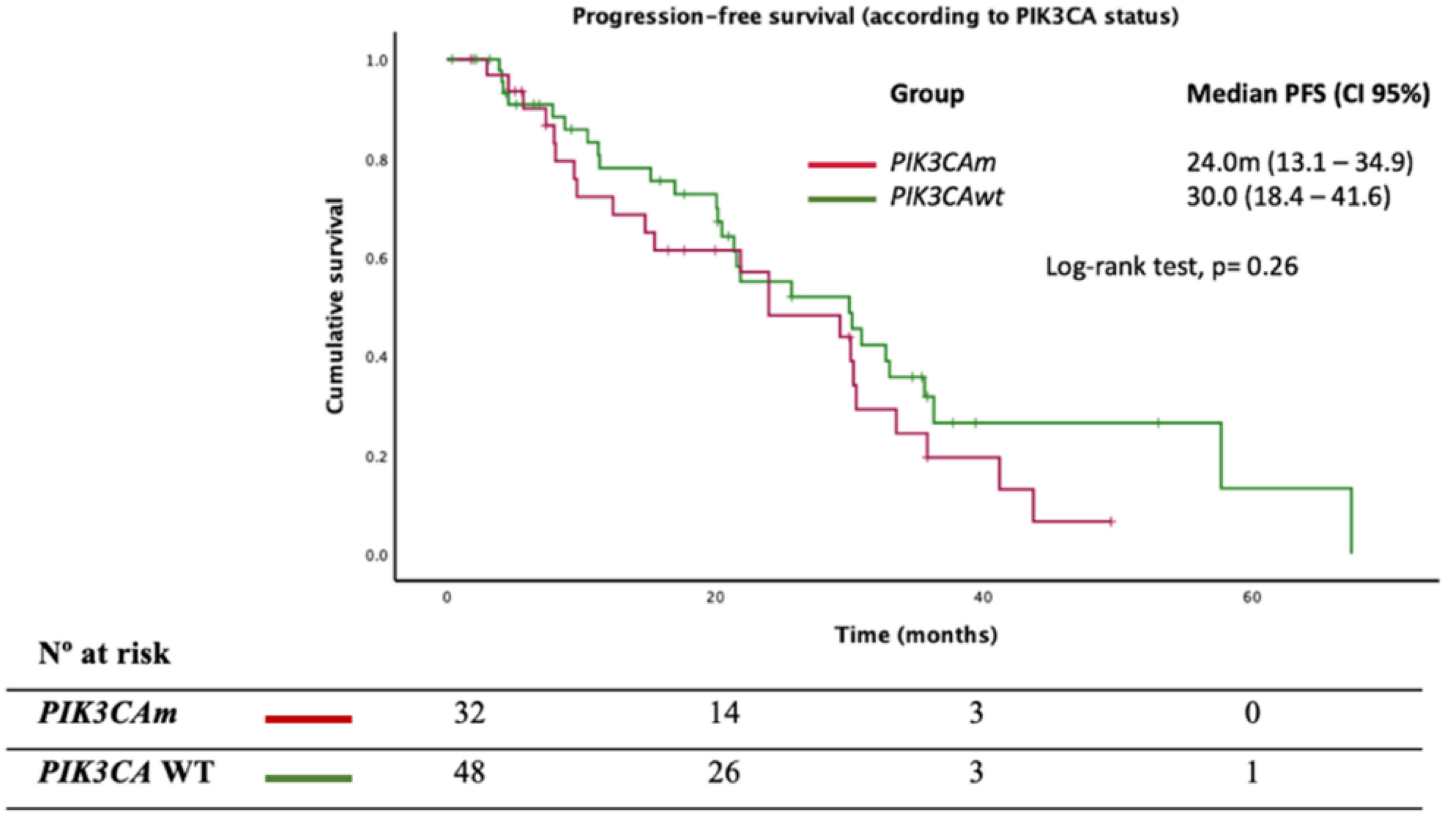
Outcomes according to progression‐free survival (PFS) in *PIK3CAm* and WT patients with cyclin inhibitor treatment.

In addition, in metastatic disease, 80 patients received treatment with CDK4/6 inhibitors + endocrine therapy: 61 in the first line and 19 in the second line. We observed reduced PFS in *PIK3CAm* carriers versus WT [24 m vs. 30 m; HR = 1.39 (95% CI, 0.7–2.4); *p* = 0.26]. Detailed information about clinical outcomes in *PIK3CAm* carriers and the WT population with cyclin inhibitors is provided in Figure 3.

We observed a similar response rate in *PIK3CAm* vs WT [68.8% vs. 77.1%, OR = 0.65 (95% CI, 0.24–1.79); *p* = 0.41]. In addition, we reported a higher proportion of visceral involvement in *PIK3CAm* versus WT [75% vs. 68.8%, OR = 1.36 (95% CI, 0.49–3.73); *p* = 0.54], and a significantly poorer PFS in patients with visceral involvement [21.6 m vs. 57.7 m; *p* = 0.001, HR = 3.6 (95% CI, 1.61–8.07); *p* = 0.002] and in those with metastatic sites ≥3 [20.2 m vs. 35.6 m, HR = 2.0 (1.13–3.59); *p* = 0.02]. Based on visceral involvement and the number of metastatic locations, we did not observe a significant prognostic impact associated with *PIK3CAm*: HR = 1.13 (95% CI, 0.63–2.04); *p* = 0.69. An extensive analysis of the prognostic impact of visceral involvement in *PIK3CAm* and WT populations treated with cyclin inhibitors is included in the Data S1.

**FIGURE 4:**
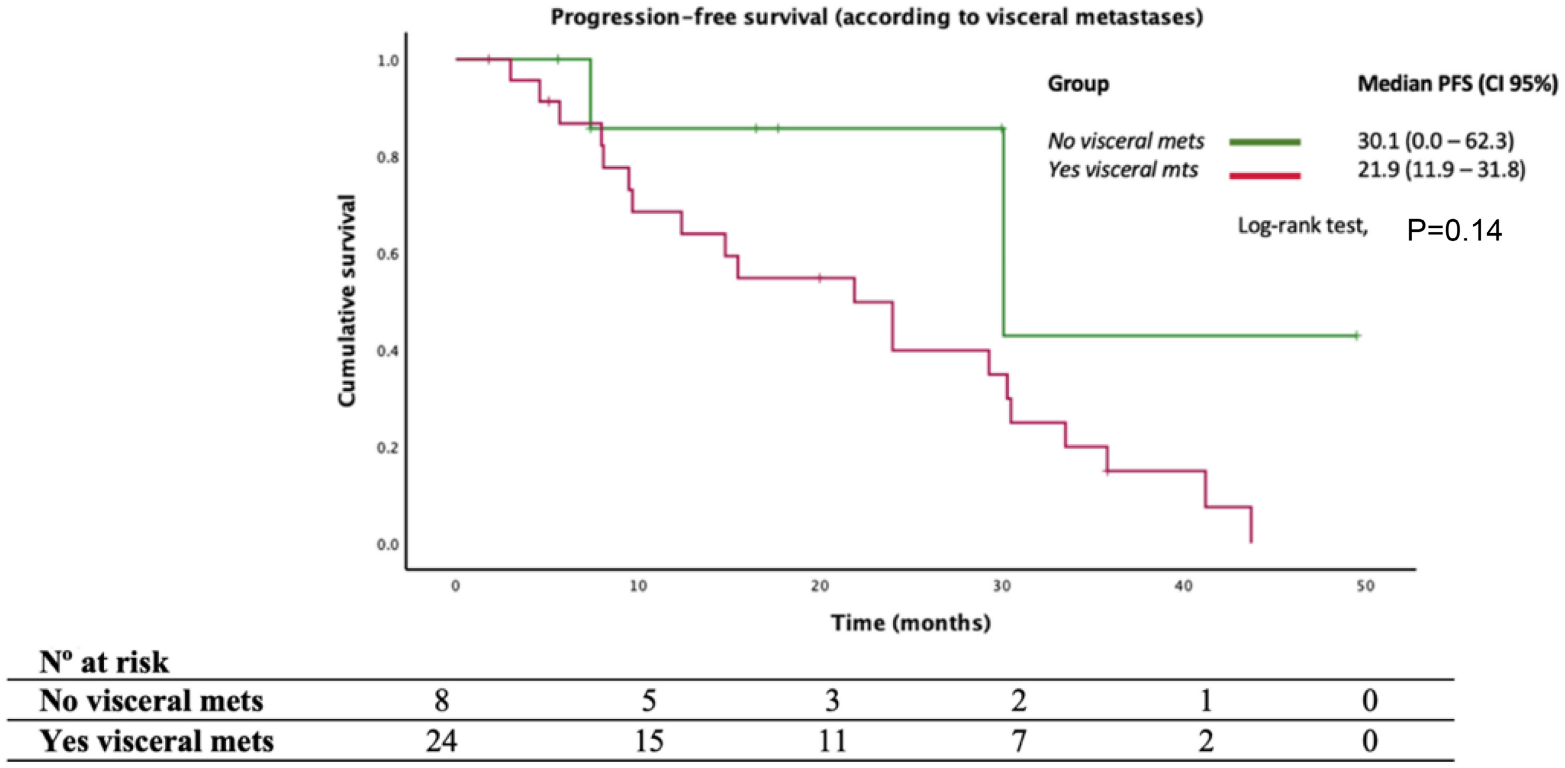
Progression‐free survival (PFS) in the *PIK3CAm* population regarding visceral involvement.

Focusing on the *PIK3CAm* population (*n* = 32), we identified worse outcomes in PFS in population with visceral involvement [21.9 m vs. 30.1 m; *p* = 0.08, HR = 2.82 (95% CI, 0.6–12.3); *p* = 0.14], as presented in Figure 4.

**FIGURE 5:**
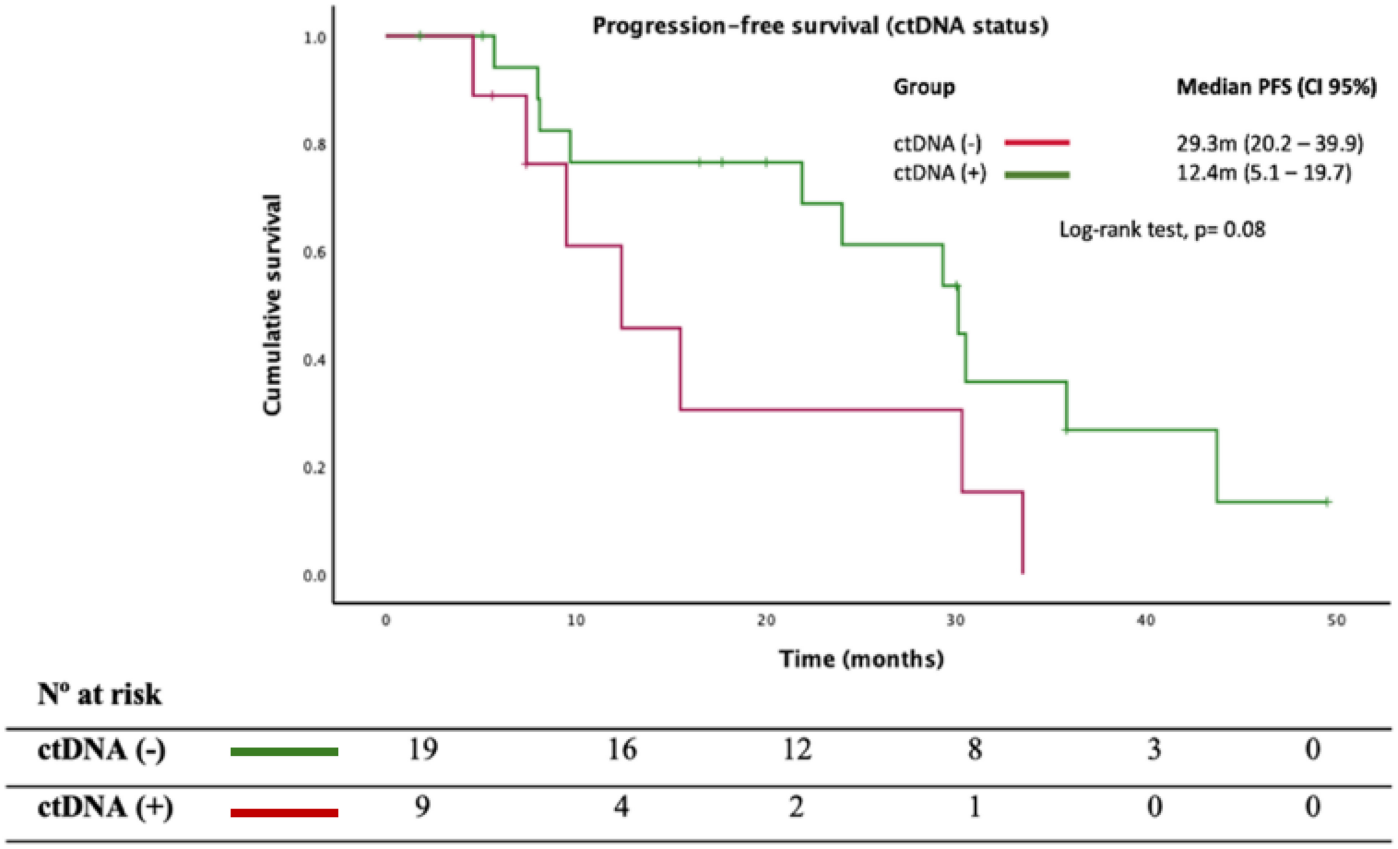
Progression‐free survival (PFS) in *PIK3CAm* regarding *PIK3CA* status in ctDNA.

In addition, *PIK3CAm* detection in plasma was related to a worse PFS compared with those cases where *PIK3CAm* was identified exclusively in tissue [12.4 m vs. 29.3 m; HR = 2.4 (95% CI, 0.8–6.5), *p* = 0.08], as reflected in Figure 5. Stratifying by visceral involvement and number of metastatic sites (≥3), we did not observe a significant prognostic impact of *PIK3CAm* in plasma; HR = 2.04 (0.59–7.02); *p* = 0.26.

In patients undergoing treatment with cyclin inhibitors, we conducted a subanalysis focusing on *PIK3CAm* in exons 9 (*n* = 15) and 20 (*n* = 12), comprising a total of 27 subjects.

**FIGURE 6:**
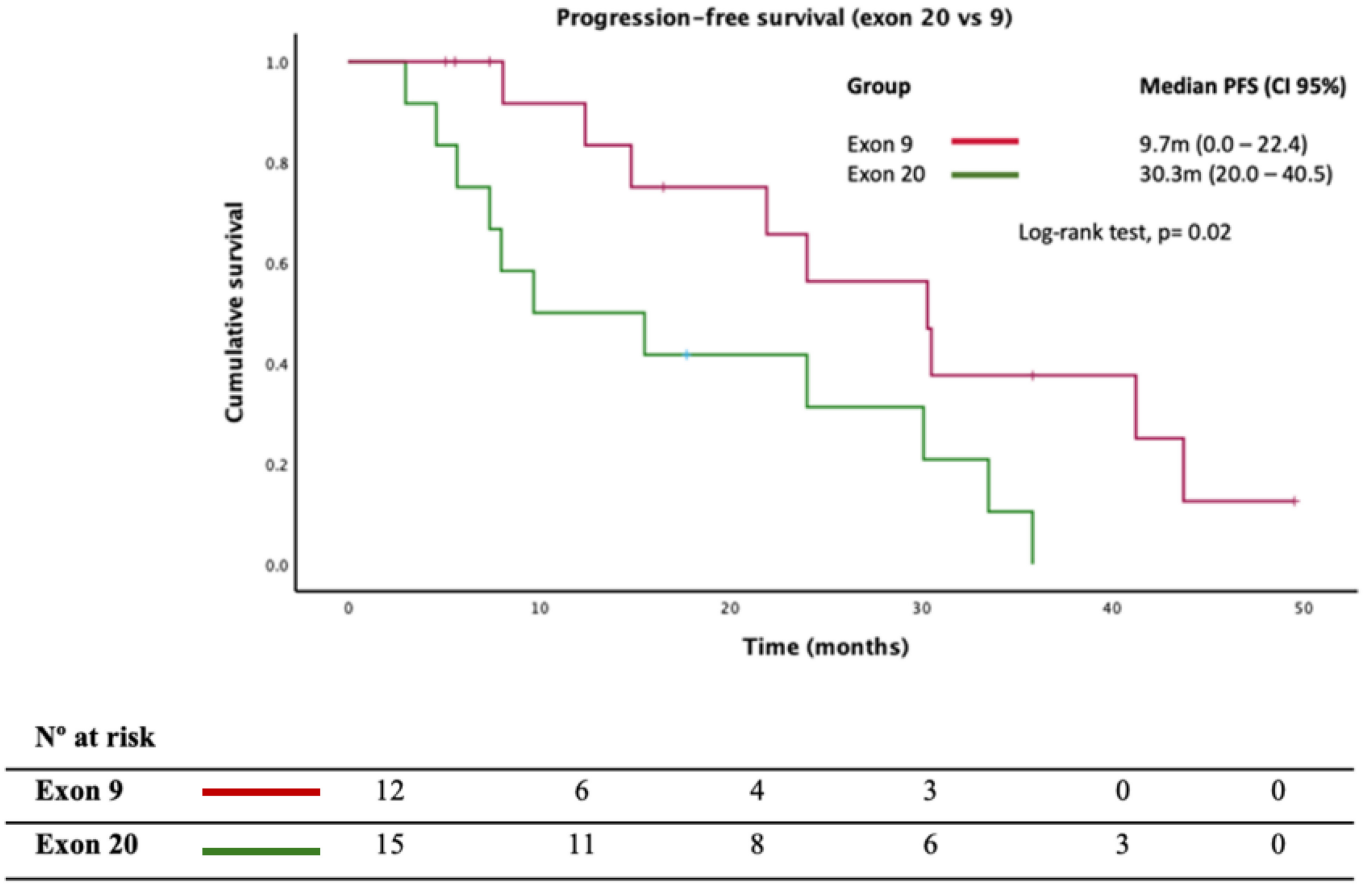
Clinical outcomes in *PIK3CAm* patients comparing exon 9 versus 20 carriers.

In 22 patients, cyclin inhibitors were used as first‐line treatment, while in 5 cases, prior exposure to endocrine therapy for advanced disease was reported. Interestingly, we observed that patients with *PIK3CAm* on exon 9 had a significantly poorer PFS versus those with *PIK3CAm* on exon 20 [9.7 m vs. 30.3 m; HR = 2.84 (95% CI, 1.1–7.4), *p* = 0.024]. The results of the exon analysis are summed up in Figure 6. No significant differences were observed in response rates between *PIK3CAm* and WT population; [73.3% vs. 75%; OR = 0.92 (95% CI, 0.16–5.21); *p* = 0.92].

Even though we reported a trend to worse PFS in patients with prior exposure to endocrine therapy in advanced disease [HR = 2.73 (95% CI, 0.87–9.32); *p* = 0.07], visceral involvement [HR = 2.92 (95% CI, 0.65–13.18); *p* = 0.16], and plasma detection of *PIK3CAm* [HR = 1.05 (95% CI, 0.33–3.61); *p* = 0.92], in a multivariate analysis, mutations in exon 9 were an independent poor prognostic factor; HR = 2.87 (95% CI, 1.02–8.55); *p* = 0.049.

## 3 DISCUSSION

In our research, we found *PIK3CAm* in 38.5% of BC (62 patients), 77.9% occurring at the three HS (E542K and E545K at exon 9 and H1047R at exon 20). Our results agree with those previously reported: *PIK3CAm* rate ranges from 30% to 40% of BC^7^ and is more frequent in HR+ disease. Similar mutation rates have been reported in advanced disease (between 30% and 40%)^15^, ^18^ and in the three HS mentioned above (70%).

Due to the clinically significant results of *PIK3CA* inhibitors in metastatic BC patients^15^ and the spatial heterogeneity of *PIK3CA* expression within BC (from primary tumor to metastases^19^), the reliable screening for *PIK3CAm* is still of paramount importance, as it could guide clinicians' decision making.

We found no significant differences in the *PIK3CAm* status (*PIK3CAm* vs. WT) between eBC and metastatic disease (38.0% vs. 38.8%; *p* = 0.91). Similarly, no differences were reported according to the biopsy location, whether from the primary tumor or metastasis. As presented in our study, there is a similar *PIK3CAm* rate in primary tumors and metastases^20^ and discordances are relatively low (approximately 10%). Along with that, Juric et al.,^21^ reported that the rate of *PIK3CA* WT eBC that changed to *PIK3CAm* is quite unusual, which could explain a stable incidence between early and late‐stage BC.

According to the screening of *PIK3CAm*, detection in fresh tumor samples and ctDNA depends on analytical and methodological reasons, which possibly affects clinical utility validation.^22^

We performed a plasma and tissue correlation study in 64 metastatic patients, with an overall correlation rate of 70.3%. It is noteworthy that 19 out of 28 patients had a discordant result: *PIK3CAm* was detected in tissue and missing in plasma.

Limited studies have explored the agreement of ctDNA vs tissue‐based methods. They show a widely variable concordance rated (26%–93%) between the two approaches^16^, ^23^, ^24^ due to low or non‐tumor shedding, technical difficulties or tumor heterogeneity.

In our study, we reported a moderate discordance rate (29%) that could be explained by the sensitivity of the technique used for ctDNA testing^25^ (Cobas®PIK3CA Kit) with an analytical sensitivity of 0.7%–3.5%.^17^ This test provides a significantly lower detection capacity than other techniques, such as exosome‐derived DNA analysis, next generation sequencing or digital PCR (detection of *PIK3CAm* below 0.1%).

Also, we observed a higher plasma correlation in patients with visceral involvement, ≥3 metastatic sites, as well as collection during disease progression, which would translate into a higher tumor burden and ctDNA.^25^ This is in line with what has been published by Oliveira et al.,^26^ who identified greater cost‐effectiveness in detecting mutations in disease progression status.

In relation to the clinicopathological characteristics, we observed a significantly higher proportion of BC with lobular histology in *PIK3CAm* population (18.1% vs. 4.1%; *p* = 0.007). Nonetheless, most studies have been carried out on invasive ductal BC, and only sporadic cases of less common histologies with better prognoses have been explored for *PIK3CA* abnormalities.^27^ Based on some reports, the frequency of *PIK3CAm* was particularly high (up to 46% of cases) in the lobular histology, with one of the highest incidences recorded so far for different types of human neoplasms.^28^

Although in our study we did not report significant differences in other clinicopathological features, we observed a trend toward smaller tumors, with less lymph node involvement and a lower Ki‐67 index. This is in line with Kalinsky et al.,^29^ who described the association with good prognostic features in *PIK3CAm* eBC, including lymph node negativity (*p* = 0.03), lower tumor stage (*p* = 0.02) and lower grade (*p* < 0.001).

In addition, *PIK3CAm* seems to display differential prognostic value in eBC; as a favorable prognostic factor^29^ but questionable by some authors,^30^ whereas, in metastatic setting, it has been strongly associated with poorer OS and resistance to chemo and endocrine therapies in advanced disease.^18^, ^31^ In the study published by Dr. Kok and colleagues,^32^ no prognostic impact of somatic *PIK3CAm* was observed in women with HR+/HER2− eBC on DFS and OS. In line with this, we did not observe in our population a higher DFS (time to metastases) in *PIK3CAm* vs WT (*p* = 0.94). According to our multivariate analysis, *PIK3CA* status did not appear to be as crucial as lymph node involvement (HR = 2.76; *p* = 0.03) or tumor grade (HR = 2.18; *p* = 0.08) in relapsing disease.

Based on the endocrine resistance conferred by *PIK3CAm*, we described in advanced disease worse PFS in *PIK3CAm* patients treated with cyclin inhibitors (21.9 m vs. 30.1 m; *p* = 0.08). This detrimental effect was greater in patients with visceral involvement and plasma detection of *PIK3CAm* and in exon 9 *PIK3CAm* carriers. Our results are along with those presented by Del Re et al.,^14^ in which they describe that *PIK3CAm* detection in liquid biopsy correlates with a poorer PFS in population with metastatic BC undergoing treatment with CDK4/6 inhibitors (7.44 vs. 12.9 months, *p* = 0.01).

However, there is still controversy regarding the prognosis associated with different *PIK3CAm*.^12^, ^13^ Our data confirm the results of Barbareschi and colleagues,^13^ in which exon 9 *PIK3CAm* were strongly related to early recurrence and death compared with exon 20 *PIK3CAm* with a better prognosis.

While these studies suggest poorer outcomes of certain *PIK3CAm* in the pre‐cyclin inhibitor era, our research supports the potential deleterious effect of mutations in exon 9 during CDK4/6 inhibitors treatment.

Therefore, this population represents an unmet clinical need. Its identification could be critical for guiding future therapeutic decisions and could be reconsidered as a potential predictive biomarker of resistance to CDK4/6 inhibitors.

In addition, a similar range of benefits has been reported with the *PIK3CA* inhibitor (alpelisib) in exons 9 and 20 *PIK3CAm* carriers, in agreement with the outcomes of the phase 3 SOLAR‐1 trial,^15^ representing a potential therapeutic option in this poorer prognosis population.

## 4 CONCLUSIONS

In conclusion, our findings suggest the *PIK3CA* evaluation in tissue as the diagnostic method of choice, however, additional investigations are warranted to better define the role of liquid biopsy in the *PIK3CA* assessment. Improvements in liquid biopsy techniques for detecting *PIK3CAm*, combined with careful timing and selection of clinicopathological characteristics, could significantly enhance detection sensitivity rates. Despite the study's limited sample size, *PIK3CAm* appear to correlate with poorer outcomes in advanced luminal BC patients treated with CDK4/6 inhibitors; significantly worse in exon 9 mutation carriers, regardless of visceral involvement and plasma detection, highlighting an urgent and unmet clinical concern. In early‐stage disease, *PIK3CAm* did not demonstrate a significant prognostic impact. These findings underscore the need for validation in a larger, prospective cohort study.

## AUTHOR CONTRIBUTIONS

**Eduardo Terán:** Conceptualization (lead); formal analysis (lead); methodology (lead). **Rebeca Lozano:** Conceptualization (lead); data curation (lead); formal analysis (lead); methodology (equal). **César A. Rodríguez:** Conceptualization (equal); data curation (equal); formal analysis (equal); project administration (lead). **Mar Abad:** Conceptualization (equal); investigation (equal); methodology (equal); project administration (equal). **Luis Figuero:** Data curation (equal); investigation (equal); supervision (equal). **José Antonio Muñoz:** Data curation (equal); investigation (equal); methodology (equal). **Belén Cigarral:** Data curation (equal); investigation (equal). **Aline Rodrígues:** Data curation (equal); investigation (equal); methodology (equal). **Magdalena Sancho:** Data curation (equal); formal analysis (equal); investigation (equal). **M. Asunción Gómez:** Formal analysis (equal); methodology (equal). **Daniel Morchón:** Data curation (equal); formal analysis (equal); methodology (equal). **Juan Carlos Montero:** Formal analysis (equal); methodology (equal); project administration (equal). **José María Sayagués:** Formal analysis (equal); methodology (equal). **M. Dolores Ludeña:** Funding acquisition (lead); investigation (equal); methodology (equal); project administration (equal). **Emilio Fonseca:** Project administration (lead).

## FUNDING INFORMATION

The project has been funded by Roche Diagnostics, S.L. within the research study “Characterization and comparison of the mutational status of the *PIK3CA* gene between the primary tumor, metastatic tissue, and peripheral blood of patients with advanced breast cancer at Complejo Asistencial Universitario de Salamanca (CAUSA) (2021–2023).”

## ETHICS STATEMENT

All procedures performed in studies with human subjects conformed to the ethical guidelines of the institutional and/or national research committee and to the 1964 Declaration of Helsinki and its subsequent modifications. The research was also endorsed by the Bioethics Committee of the University Hospital of Salamanca.

## CONSENT

In addition, informed consent was provided by the subjects involved in the study. All procedures were conducted according to the appropriate standards. Also, all participants in this study have consented to the publication of this manuscript.

## Supporting information


Data S1:

## Data Availability

Data sets used and analyzed during the present study are available from the corresponding author upon reasonable request to safeguard the confidentiality of the patient's clinical data.
