# Supplementary material for: PIK3CA mutational status in tissue and plasma as a prognostic biomarker in HR+/HER2− breast cancer
Source: Cancer Med. 2024 Sep 5;13(17):e70101. doi: 10.1002/cam4.70101 (PMC11375731; doi:10.1002/cam4.70101)
Supplement: Supplementary file 1 — Data S1: [file CAM4-13-e70101-s001.docx]

Supplementary Material

1. **Technical analysis of *PIK3CA* in plasma and tissue (Material and Methods):**

To obtain cfDNA from liquid biopsy, 20 mL of peripheral blood was extracted into EDTA tubes for each patient. Blood plasma was obtained by centrifugation as follows: The tubes containing the blood were centrifuged at 2000 × g for 10 minutes at 4ºC. Afterwards, the supernatant was recovered and placed in a new tube (5ml), then centrifuged at 16,000 x g for 10 minutes at 4ºC. The resulting supernatant was aliquoted into new tubes in 2 ml aliquots. An aliquot containing 2 ml of plasma obtained from each patient was used to isolate cfDNA using a Cobas® cfDNA Sample Preparation Kit (Roche, Branchburg, NJ, USA) and following the manufacturer’s instructions.

To obtain DNA from paraffin-embedded tissue, a paraffin block containing at least 70% tumor cells was used. One or two 10 μm-thick formalin-fixed paraffin-embedded (FFPE) tumor tissue sections were deparaffinized with xylene for 5 minutes at room temperature (RT), dehydrated in absolute alcohol for 5 minutes at RT, and allowed to air-dry completely for 10 minutes. Subsequently, DNA was isolated using the Cobas DNA Sample Preparation Kit (Roche, Branchburg, NJ, USA) following the manufacturer’s instructions.

The DNA obtained from FFPE and liquid biopsy samples were used to analyze 17 mutations across exons 1, 4, 7, 9, and 20 of the *PIK3CA* gene (Roche Diagnostics) using the Cobas® PIK3CA Kit (Cobas® platform, Roche Diagnostics).

1. **Evaluation of *PIK3CA* in population treated with cyclin inhibitors (Results):**

Analysis of the prognostic impact of visceral involvement in *PIK3CAm* and *PIK3CA* WT populations treated with cyclin inhibitors. We reported a trend towards a higher proportion of visceral involvement in the *PIK3CAm* vs WT population (75% vs. 68.8%); p=0.54. The proportion of visceral involvement according to *PIK3CA* status is summarized in Table S1.

|  |  | **Visceral involvement** | |  |  |
| --- | --- | --- | --- | --- | --- |
|  |  | **No (%)** | **Yes (%)** | **Total (%)** |  |
| ***PIK3CA* status** | ***PIK3CA* WT** | 15 (31.3%) | 33 (66.8%) | 48 (100%) |  |
|  |  |  |  |  |  |
|  | ***PIK3CAm*** | 8 (35.0%) | 24 (75.0%) | 32 (100%) |  |
|  |  |  |  |  |  |
|  | **Total** | 23 (28.7%) | 57 (71.3%) | 80 (100%) |  |

**Table S1.** Proportion of patients with visceral involvement of according to *PIK3CA* status.


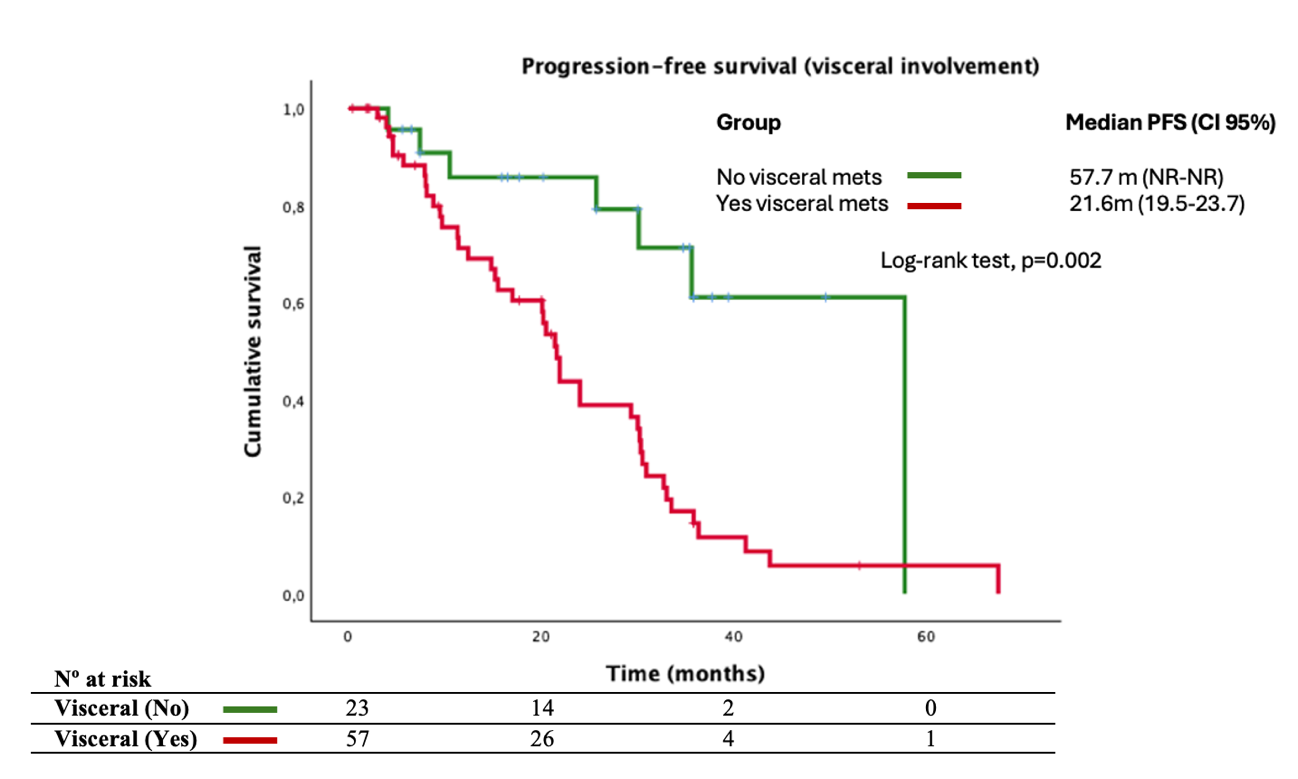
Those pts with visceral involvement exhibited a significantly shorter PFS (21.6m vs 57.7m; p=0.001, HR=3.6 (1.61-8.07); p=0.002), as reflected in Figure S1.

**Fig S1.** PFS in the population treated with cyclin inhibitors according to the presence or absence of visceral involvement.
